# High-Throughput Rice Density Estimation from Transplantation to Tillering Stages Using Deep Networks

**DOI:** 10.34133/2020/1375957

**Published:** 2020-08-21

**Authors:** Liang Liu, Hao Lu, Yanan Li, Zhiguo Cao

**Affiliations:** ^1^National Key Laboratory of Science and Technology on Multi-Spectral Information Processing, School of Artificial Intelligence and Automation, Huazhong University of Science and Technology, Wuhan, 430074 Hubei, China; ^2^The University of Adelaide, Adelaide, SA 5005, Australia; ^3^School of Computer Science and Engineering, Wuhan Institute of Technology, Wuhan, 430205 Hubei, China

## Abstract

Rice density is closely related to yield estimation, growth diagnosis, cultivated area statistics, and management and damage evaluation. Currently, rice density estimation heavily relies on manual sampling and counting, which is inefficient and inaccurate. With the prevalence of digital imagery, computer vision (CV) technology emerges as a promising alternative to automate this task. However, challenges of an in-field environment, such as illumination, scale, and appearance variations, render gaps for deploying CV methods. To fill these gaps towards accurate rice density estimation, we propose a deep learning-based approach called the Scale-Fusion Counting Classification Network (SFC^2^Net) that integrates several state-of-the-art computer vision ideas. In particular, SFC^2^Net addresses appearance and illumination changes by employing a multicolumn pretrained network and multilayer feature fusion to enhance feature representation. To ameliorate sample imbalance engendered by scale, SFC^2^Net follows a recent blockwise classification idea. We validate SFC^2^Net on a new rice plant counting (RPC) dataset collected from two field sites in China from 2010 to 2013. Experimental results show that SFC^2^Net achieves highly accurate counting performance on the RPC dataset with a mean absolute error (MAE) of 25.51, a root mean square error (MSE) of 38.06, a relative MAE of 3.82%, and a *R*^2^ of 0.98, which exhibits a relative improvement of 48.2% w.r.t. MAE over the conventional counting approach CSRNet. Further, SFC^2^Net provides high-throughput processing capability, with 16.7 frames per second on 1024 × 1024 images. Our results suggest that manual rice counting can be safely replaced by SFC^2^Net at early growth stages. Code and models are available online at https://git.io/sfc2net.

## 1. Introduction

Plant counting is a fundamental task in agriculture. It is an important index for crop growth monitoring. For example, the total number of maize tassels determines whether maize plants step into the tasseling stage [1]. The number of root nodules is an indicator of the health status of soybean [2]. The dynamics of the pest population [3] benefits pest forecasting. In addition, knowing the condition of the weeds helps farmers to spray herbicide and to optimize its use [4]. More importantly, many counting results are closely related to the crop yield, such as the number of wheat ears per unit ground area (ear density) [5, 6] and the number of fruits [7].

Rice is one of the most important cereal crops in the world. Its annual production is more than 590 million tons [8]. The numbers of leaves [9], panicles [10], spikes [11], and particles [12] are common statistic indexes of interest. In particular, rice density is closely associated with cultivated area statistic and management [13], as well as how to maximize the use of cultivated land [14]. The increase in planting density can suppress the growth of weed [15] and improve the efficiency of nitrogen and the yield of rice [16]. In addition, the number of survival rice plants is one of the key metrics in rice breeding [17]. It is also related to damage evaluation caused by typhoon [18] and flood [19].

**Figure 1:**
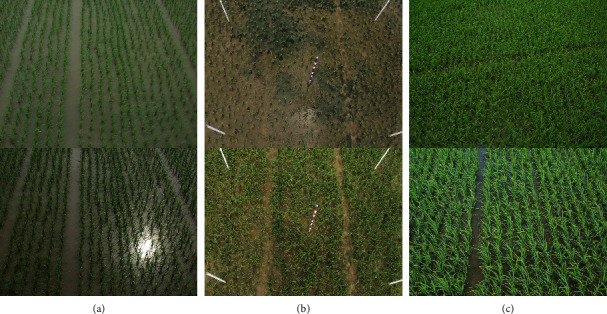
Some examples showing the difficulties in rice counting: (a) shows illumination variations, (b) shows the appearance changes when rice grows, and (c) shows overlapping scenes.

Nowadays, in-field rice plant counting still depends on manual sampling and statistics. Agricultural observers must frequently process each plant manually, which is tedious, inefficient, and inaccurate. Such an observation manner easily causes irreversible damage to rice. With the prevalence of a low-end digital camera, computer vision technology emerges as a potential automated solution. However, it faces many difficulties shown in Figure 1:

- *Occlusions*. Since the camera is fixed, rice that is far from the camera tends to be blocked by the one that is close to the camera. Neighboring leaves may occlude rice planted in the same row.
- *Illumination Variations*. The illumination of the outdoor environment changes significantly because of the changing sunlight. Since rice grows in a field covered by water, the water leads to sunlight reflection, thus rendering unreliable imagery.
- *Appearance Variations*. The appearance of rice plants varies at different growth stages. For example, the height and stem diameter of rice at the returning green stage increase obviously compared with those at the transplant seeding stage.
- *Scale Differences*. A fixed camera also generates perspective distortion. Objects close to the camera are large in the visual field. Even for the same rice plant, images taken at different heights show different sizes.

The problems above not only appear in rice counting but also are pain spots in generic object counting in CV. Many effective CV-based counting approaches have been developed to address these issues. For example, occlusions can be alleviated in the density regression-based paradigm [20]. Appearance variations caused by illumination or different growth stages can be mitigated via a multicolumn feature encoder [21], a pretrained model [22], or a feature fusion strategy [23]. Further, scale variations often lead to sample imbalance. This problem is well addressed by transforming count values to quantized count intervals (counting class) [24]. Hence, we propose to integrate these successful counting ideas into SFC^2^Net for rice density estimation. First, in the feature extraction stage, SFC^2^Net introduces ImageNet-pretrained MixNet-L [25] as the backbone (a multicolumn light-weight convolution architecture) to enhance feature representation. Second, multilayer fusion is used to fuse feature maps from different convolution layers to further increase appearance robustness. Third, following [24, 26], a redundant module generates a redundant count interval map (class map) to address object splitting and sample imbalance. Finally, in the inferring stage, the redundant class map is normalized by inverse quantization [24] and deredundancy [26] to generate a count map. The final count of the input image can thus be computed by summing over the normalized count map.

We evaluate our method on a middle-scale rice plant counting (RPC) dataset, which includes 382 high-resolution images. They are collected from two field sites in China from 2010 to 2013. We manually annotate dots for each plant. Extensive experiments verify the effectiveness of each module and report highly accurate counting performance on the RPC dataset with a mean absolute error (MAE) of 25.51, a mean square error (MSE) of 38.06, a relative MAE of 3.82%, and a *R*^2^ of 0.98. In addition, SFC^2^Net can process 1024 × 1024 images with 16.7 frames per second (FPS), implying its high-throughput nature.

Overall, we make the following contributions:

- We integrate several successful object counting ideas and present a novel deep learning-based rice counting approach, SFC^2^Net, for rice density estimation
- We introduce a new rice plant counting (RPC) dataset with dotted manual annotations
- We show that traditional manual rice counting can be safely replaced with our automated solution presented in this work

## 2. Related Work

We review recent counting methods in computer vision and their applications in crop phenotyping.

### 2.1. Object Counting in Computer Vision

Early object counting methods in CV are derived from object detection where each object is detected by a hand-crafted feature classifier [27], and the number of the detected objects is summed to be the object counts. Another solution is with the help of video [28], which first segments the foreground and background by motion information and then sums the foreground objects. Considering that bounding box- and pixel-level annotations are expensive, a milestone work [20] translates counting into density map regression. At this time, another regression solution [29] is to regress the local count.

After deep learning achieves unprecedented success [30], it is introduced into the counting community. [31] is the first work applying deep learning to counting by estimating the density map and object counts simultaneously. Ever since, deep learning-based methods become popular for object counting. According to different learning targets, typical deep learning-based methods can be classified into the following paradigms: density map-based method, count map-based method, class map-based method, dot map-based method, and detection-based method. Density map estimation is still the mainstream which inherits from traditional methods. [21, 32] utilize a multicolumn convolution neural network (MCNN), where different columns have convolutional layers of different receptive fields to increase size robustness of objects. [22] uses VGG16 [33] as its backbone and dilated convolution to increase the receptive field. Furthermore, aside from density map-based methods, count map-based methods are also developed where each point represents the count of a block in the input image. In this paradigm, [26] regresses patch count for each patch independently, while [34] uses a fully convolutional network for estimation. In order to solve the problem of sample imbalance in count map-based methods, count values are converted to count intervals through nonlinear quantization [24, 35], thereby transforming a counting task into a blockwise multiclass classification problem. Besides, a dot map-based method [36] is proposed to compute the dot map directly without the help of the Gaussian kernel. Apart from these map-based methods, with the development of weakly supervised object detection, detection-based counting methods return to the eye of researchers. For example, [37] extracts bounding box information from dotted annotations to train a crowd detection network. In summary, CV-based counting methods have the following advantages:

- The architecture of the fully convolutional network (FCN) pretrained on ImageNet can extract powerful and descriptive features with high efficiency
- The multiscale feature encoder (typically MCNN) can extract multigrained features and further improve the feature description
- By quantizing counts into count intervals, counting models can alleviate sample imbalance caused by scale variations

SFC^2^Net inherits several key advantages from object counting networks. It extracts multiscale features by a multibranch ImageNet-pretrained MixNet-L in a fully convolutional manner. It also predicts a redundant class map to alleviate sample imbalance.

### 2.2. Plant Counting in Crop Phenotyping

Recently, some CV-based methods have been proposed for plant counting, which can be classified into traditional methods and deep learning-based methods. The traditional methods commonly segment plants or detect them by hand-crafted low-level features and count the detected objects. For example, [7] segments apples by a threshold, which is further dealt by morphological processing to identify the count of apples. [38] extracts the SIFT descriptors from superpixels and trains a support vector machine to classify the fruit and nonfruit areas. On the contrary, deep learning-based detection methods employ a data-driven network for segmentation/detection. For instance, [39] utilizes Faster RCNN [40] to detect wheat spikes. Another deep learning-based counting paradigm is to employ CNNs to infer the count from an image directly. In this paradigm, [41] regresses the global count from images captured by drones. TasselNet [26] introduces local patch regression into maize tassel counting. Further, [42] combines density map regression and background segmentation to estimate the count of rice seedlings. However, current crop counting methods have the following points that can be further improved:

- Detection/segmentation-based methods tend to fail when tackling partially overlapping objects
- Regression-based methods suffer from sample imbalance, which is caused by inhomogeneous distribution and gives rise to a training bias
- For traditional methods, they are unable to adapt to complex scenes in real-world scenarios because the features are not strong enough
- For deep learning-based methods, they commonly use a simple structure of the backbone, which limits the feature expression to scale variations

SFC^2^Net overcomes these disadvantages with a carefully chosen feature backbone, a well-designed feature fusion strategy, and a delicately developed learning paradigm.

## 3. Materials and Methods

### 3.1. Experimental Fields and Imaging Devices

**Figure 2:**
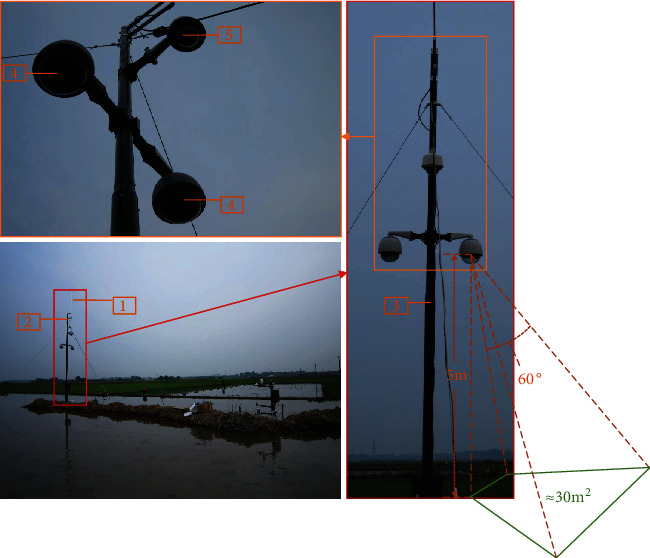
Image acquisition device in the rice field. The numbers in the image represent the following: 1—lighting rod, 2—antenna, 3—support, 4—CCD cameras, and 5—monitoring camera.

The experimental field images analyzed in this work were captured in Jiangxi and Guangxi Provinces, China. All the images were taken under natural illumination from 2010 to 2013. The imaging device includes an image-capturing and a communication system [43], as shown in Figure 2. The image-capturing system is used for data collection. In detail, rice image sequences (4272 × 2848) are captured in Jiangxi with an OLYMPUS E-450 camera during the daytime from 9:00 to 16:00 every hour within 2010 to 2013. Similarly, rice images with resolution 3648 × 2736 are also captured in Guangxi with a Canon EOS 1100D camera during the daytime from 7:00 to 19:00 every hour within 2012 to 2013. Moreover, the communication system including the antenna and encoder is used for data transmission through 3G wireless networks.

### 3.2. Rice Plant Counting Dataset

We choose 382 high-resolution in-field rice images from 10 image sequences (two sequences in Jiangxi from 2010 to 2013 and one sequence in Guangxi from 2012 to 2013) from the transplantation stage to the tillering stage. The rice sizes vary from 80 × 80 pixels to 300 × 300 pixels, with spatial resolutions ranging from 1.51 mm^2^/pixel to 5.65 mm^2^/pixel. Considering that there were more than one thousand plants in the images taken in Jiangxi from April 2012 to May 2012 only, we divide the first half of them in the training set and the rest in the test set. Overall, the rice plant counting dataset consists of 230 training images and 152 testing images.

**Figure 3:**
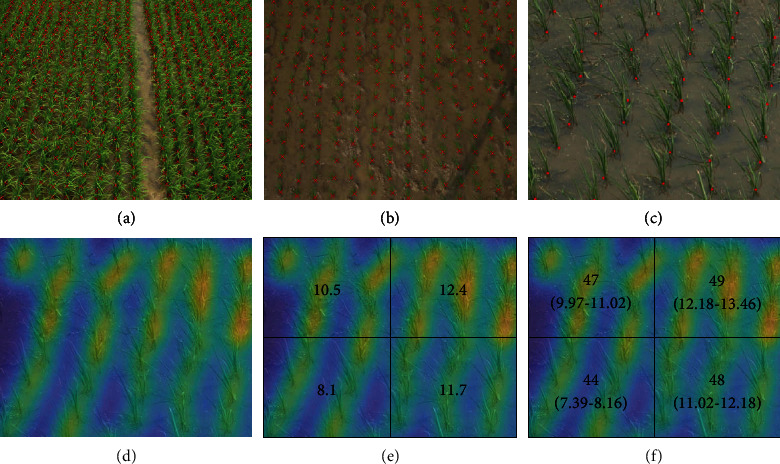
Annotation samples (a and b, the cross center is the labeled point for rice root) and conceptual differences between a dot map (c), density map (d), count map (e), and class map (f).

Following the standard counting annotation paradigm, we manually mark a point at the root of each plant. Indeed, point annotations are considered the most natural way to count objects, especially for dense objects, because the burden of point annotations is less than that of other fine-grained annotations such as bounding boxes or pixels.  Figure 3 shows some samples with dotted annotations. In our training set, the maximum count of an image is 1330, the minimum is 182, and the average is 493.11. In our testing set, the maximum count of an image is 1255, the minimum is 341, and the average is 648.39. The total number of annotations in the RPC dataset is 211,971.

### 3.3. Learning Target

Here, we describe the learning target of the model because this learning target is not in accordance with one's common sense. Differing from the local patch regression task that estimates the count map directly [26, 44], in this paper, blockwise classification counting was introduced [24], which estimates the class map describing the counting intervals. The reason why we use blockwise classification counting is that, by quantizing the patch count into the counting interval via nonlinear transformation, it can ameliorate the sample imbalance [24].

We show how to generate the class map from dotted annotations step by step. An example demonstrating the differences between the dot map, density map, count map, and class map is shown in Figure 3. Following the standard counting paradigm, a density map is first obtained from the dot map [20]. This process can be defined by

(1) $$\begin{array}{c} D\left(i\right)=\underset{i\in P}{\sum }N\left(i;P,\sigma^{2}\right), \end{array}$$

where *i* ∈ *I* is the pixel in image *I*, *P* is the dot map of *I* by setting the pixel of annotated points to be 1 (otherwise 0), and *𝒩*(*i*; *P*, *σ*^2^) is the 2-D Gaussian kernel parameterized by *σ*. This equation is equivalent to a convolution operation on the dot map with the Gaussian kernel.

Given the density map, a count map is further computed by blockwise summation [26], defined by

(2) $$\begin{array}{c} N\left(b_{j}\right)=\underset{k\in b_{j}}{\sum }D\left(k\right), \end{array}$$

where *b*_*j*_ is the *j*-th block in *I* and *k* ∈ *b*_*j*_ is the pixel within *b*_*j*_. To train a local count regression model, *ℓ*_1_ loss can be used, which takes the following form:

(3) $$\begin{array}{c} \ell_{1}=-\underset{j\in I}{\sum }\left|N\left(b_{j}\right)-N^{\text{gt}}\left(b_{j}\right)\right|, \end{array}$$

where *N*^gt^(*b*_*j*_) is the ground truth count of patch *b*_*j*_.

Given the count map, following [24], we further quantize the count map to obtain the class map *C*(*b*_*j*_) by

(4) $$\begin{array}{c} C\left(b_{j}\right)=\left\{\begin{array}{cc} 0, & N\left(b_{j}\right)=0,\\ C_{t}\left(N\left(b_{j}\right)\right), & \text{otherwise}, \end{array}\right. \end{array}$$

where

(5) $$\begin{array}{c} C_{t}\left(N\right)=\max\left(\text{floor}\left(\frac{\log\left(N\right)-q}{s}+2\right),1\right), \end{array}$$

where *s* is the quantization step and *q* is the start of the log space. log(0) is excluded where the majority of samples are the background. To quantize all patch samples, background patches and the patches whose count values are between 0 and *e*^*q*^ are set to be independent classes. After quantization, we transform local count regression into blockwise classification. To train a multiclass classification model, the cross-entropy loss is used, defined by

(6) $$\begin{array}{c} \ell_{c}=-\underset{j\in I}{\sum }\overset{C_{\mathrm{Max}}}{\underset{c=0}{\sum }}\left(c==C_{\text{gt}}\left(j\right)\right)\log p\left(j,c\right), \end{array}$$

where *p*(*j*, *c*) is the probability of the *j*-th block for the *c*-th counting interval, *C*_Max_ is the maximum counting interval, and *C*_gt_(*j*) is the ground truth counting interval of the *j*-th block.

At the inferring stage, to recover the count map from the class map, the median of each interval is set as its count value [24], i.e.,

(7) $$\begin{array}{c} N\left(C\left(b_{j}\right)\right)=\left\{\begin{array}{cc} 0, & C\left(b_{j}\right)=0,\\ \frac{N_{t}\left(C\left(b_{j}\right)\right)+N_{t}\left(1+C\left(b_{j}\right)\right)}{2}, & \text{otherwise}, \end{array}\right. \end{array}$$

where

(8) $$\begin{array}{c} N_{t}\left(C\right)=\left\{\begin{array}{cc} 0, & C\leq 1,\\ e^{q+\left(C-2\right)\times s}, & \text{otherwise}, \end{array}\right. \end{array}$$

and *C*(*b*_*j*_) is the estimated count interval of block *b*_*j*_.

### 3.4. Overview of SFC^2^Net

**Figure 4:**
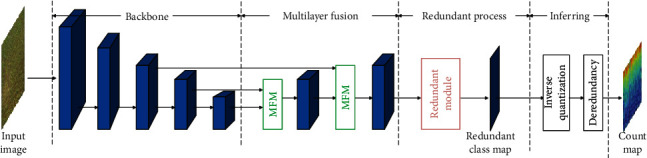
Overview of SFC^2^Net. The MixNet-L backbone first extracts feature maps that are further fused by multilayer fusion modules (MFM). Then, the redundant module processes multiscale feature maps to generate a redundant class map. Finally, after inverse quantization and deredundancy, SFC^2^Net outputs the count map. The final count of the input image is computed by summing each pixel in the count map.

As shown in Figure 4, SFC^2^Net includes four parts: a MixNet-L backbone, a multilayer fusion module, a redundant processing module, and an inferring module. To compute the number of rice plants in the image, multiscale feature maps are first extracted by the MixNet-L backbone and then fused by a multilayer fusion module. The fused feature maps are subsequently processed by the redundant module to compute the redundant (overlapping) class map. During the inference process, inverse quantization and deredundancy modules convert the redundant class map into a count map. The total count of the image can be calculated by simply summing the count map. We describe each module in detail next.

### 3.5. MixNet-L Backbone

**Figure 5:**
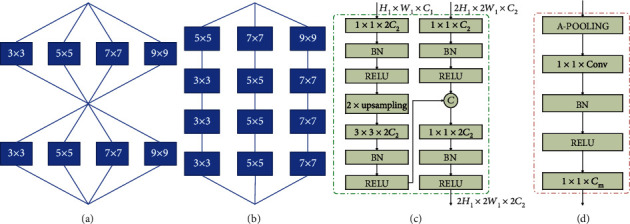
Structure diagrams. (a) is a typical structure of MixNet while (b) is that of multicolumn CNN. Common modules (single group convolution and pooling) are not shown. (c) Multilayer fusion (MFM). The input of the higher layer is the *H*_1_ × *W*_1_ × *C*_1_ feature map, and the input of the lower layer is 2*H*_1_ × 2*W*_1_ × *C*_2_. The output of this model is the 2*H*_1_ × 2*W*_1_ × 2*C*_2_ feature map. “BN” denotes batch normalization. “*C*” means the concatenation operator. (d) Redundant module. “A-POOLING” means average pooling. “*C*_*m*_” denotes the number of counting intervals.

A multicolumn network [21] is a popular solution to mitigate scale variations and increase the feature description. In this architecture, convolution kernels of different sizes in different columns extract multiscale feature maps to take object size variations into account. In this work, we introduce MixNet [25] into our model. It also utilizes filters with different receptive fields and is proven to be a powerful backbone in the ImageNet [33] classification. The typical structural difference between MCNN and MixNet is shown in Figure 5. MCNN fuses multiscale feature maps only once before the count map estimation, while MixNet fuses the feature maps after each multikernel convolution (“group convolution”). According to different application scenarios, MixNet has three types of architectures called MixNet-S, MixNet-M, and MixNet-L, respectively. MixNet-S has few layers than MixNet-M and MixNet-L, while MixNet-L has the identical architecture with MixNet-M but is with extra convolution channels. In this work, we select MixNet-L as our backbone.

### 3.6. Multilayer Fusion

The multilayer fusion module (MFM) is used to fuse the multilayer feature map to enhance the feature representation further. The structure of this module is shown in Figure 5. In each step, the decoder fuses features from two adjacent layers and outputs a fused feature map. In this module, channels of the feature map in the high-level layer are adjusted to be 2 times larger than those in the low-level layer. Next, it is upsampled by bilinear interpolation and further concatenated with the low-level feature map. The reason for adjusting feature map channels is to highlight high-level features, which include high-level semantic information, and to make it play the major role in the fused feature map. Low-level features are only treated as auxiliary information that supplements details. By using this module, all feature maps from each layer can be fused as long as the feature channels are changed accordingly. In this work, we only fuse three layers. We also investigate how to choose the number of fusion layers in experiments.

### 3.7. Generating a Redundant Class Map

In [26], to ameliorate the effect that block splits an object, a patch with overlap (the patch size is 32 × 32 and the stride is 8) was sampled and the count from overlapped patches was averaged. Following [26], a similar redundant module is concatenated after MFM for redundant evaluation. The structure of the redundant module is shown in Figure 5. First, an average pooling layer processes the feature map to generate the redundant feature maps. As mentioned above, we fuse three high-layer features, whose minimum downsample rate is 8. Thus, the downsampling rate for the feature map after the fusion stage is 8 (the patch size is 8 × 8 and the stride is 8). To be the same with the setting in [26], the kernel size of average pooling layers is 4 and the stride is 1.

After average pooling, the model outputs the response map for each counting interval via a 1 × 1 × *C*_*m*_ convolution, which further generates a probability map after a SoftMax layer. The probability map further generates the redundant class map by selecting the class interval with the maximum response. Training loss (Equation (6)) is generated from here to increase the probability of the ground truth counting interval.

### 3.8. Deredundancy

**Figure 6:**
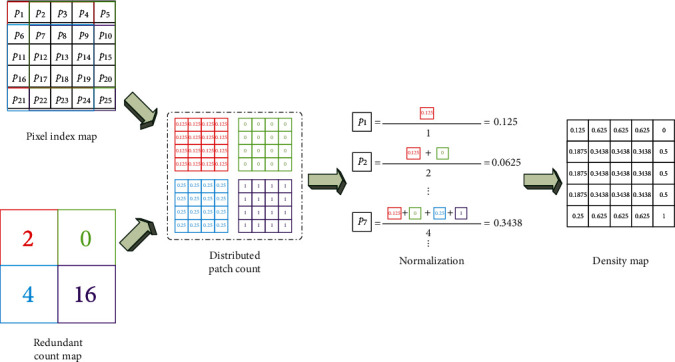
The detailed example of deredundancy.

In this section, we explain the deredundancy process that decodes the redundant class map. First, the patch count is distributed evenly across each pixel it contains as

(9) $$\begin{array}{c} D_{b_{j}}^{d}\left(x_{j},y_{j}\right)=\frac{N\left(b_{j}\right)}{n_{b_{j}}}, \end{array}$$

where (*x*_*j*_, *y*_*j*_) ∈ *b*_*j*_ are the pixels within block *b*_*j*_ whose total number of pixels is *n*_*b*_*j*__ and *N*(*b*_*j*_) is the estimated patch count of *b*_*j*_. The final count map is computed by pixel-level average normalization, defined by

(10) $$\begin{array}{c} D^{\text{o}}\left(x,y\right)=\frac{\sum_{b_{j}}D_{b_{j}}^{d}\left(x,y\right)}{T\left(x,y\right)}, \end{array}$$

where *T*(*x*, *y*) denotes the computing times of pixel (*x*, *y*) in the output count map *D*^o^. A detailed example of deredundancy is shown in Figure 6.

### 3.9. Implementation Details

We implement our method based on PyTorch [45, 46]. The initial parameters of the backbone network are loaded from the ImageNet- [30] pretrained MixNet-L. Other parameters are initialized by the Xavier method [47]. To reduce computational consumption, we downsample the original high-resolution images to their 1/4 resolution. When training a model, we randomly crop 384 × 384 patches (each image generates one cropped patch in each training epoch) from the downsampled image. Images are preprocessed by mean subtraction and division of standard deviation (the mean and standard deviation are calculated from the training set). We employ stochastic gradient descent (SGD) to optimize the model. The batch size is set to 8. The initial learning is set to 1*e*^−2^ and is decreased by a factor of 10 every 200 epochs. We train the model for 600 epochs.

## 4. Results

In this section, we show extensive experiments for SFC^2^Net on the RPC dataset. First, we introduce the evaluation metrics. Second, some ablation studies are presented to show the effectiveness of the designed modules. Third, our method is compared with some state-of-the-art counting approaches. Unless otherwise noted, the model leverages MixNet-L as our backbone and fuses 3-layer feature maps. In addition, the default quantization parameters are *s* = 0.1 and *q* = −2, and the default Gaussian kernel is set to 4. We hypothesize that each hyperparameter is independent of each other.

### 4.1. Evaluation Metric

The mean absolute error (MAE) and root mean square error (MSE) are the standard metrics for object counting which are defined as follows:

(11) $$\begin{array}{c} \text{MAE}=\frac{1}{N}\overset{N}{\underset{n=1}{\sum }}\left|\text{gt}\left(n\right)-\text{est}\left(n\right)\right|, \end{array}$$

(12) $$\begin{array}{c} \text{MSE}=\sqrt{\frac{1}{N}\overset{N}{\underset{n=1}{\sum }}{\left|\text{gt}\left(n\right)-\text{est}\left(n\right)\right|}^{2}}, \end{array}$$

where *N* denotes the total number of test images, gt(*n*) is the ground truth count of image *n*, and est(*n*) is the inferred count. The performance of MAE shows the accuracy while MSE shows the estimating stability. In addition, relative MAE (rMAE) is also used in evaluation, defined by

(13) $$\begin{array}{c} \text{rMAE}=\overset{N}{\underset{n=1}{\sum }}\frac{\left|\text{est}\left(n\right)-\text{gt}\left(n\right)\right|}{\text{gt}\left(n\right)}\times 100\%. \end{array}$$

### 4.2. Ablation Study

#### 4.2.1. Blockwise Classification versus Local Count Regression

Here, we compare blockwise classification with local count regression for rice plant counting. To adapt our architecture to regression, the final 1 × 1 × *C*_*m*_ convolution in the redundant module is replaced with a 1 × 1 × 1 convolution kernel. In this paradigm, the training target is changed back to local patch counting, and *ℓ*_1_ loss (Equation (3)) is leveraged to train the model. Thus, the output of this model is a redundant count map, which is further processed by deredundancy to output the count map as in [44]. The results shown in Table 1 illustrate that the blockwise classification counting obviously reduces the MAE by more than 25% compared with the regression baseline.

#### 4.2.2. Backbone Comparison

Here, we verify the effectiveness of the MixNet-L backbone. We compare MixNet-L with VGG16 [33], which is widely used in crowd counting and has shown good performance and generalization [22]. We compare the performance by replacing the backbone of SFC^2^Net. The results shown in Table 2 illustrate the advantage of the MixNet-L backbone. In particular, MixNet-L reduces the MAE and MSE by more than 15% and 34%, respectively, compared with VGG16 in our method. This experiment verifies the effectiveness of the MixNet-L backbone.

#### 4.2.3. Sensitivity of Model Parameters

**Figure 7:**
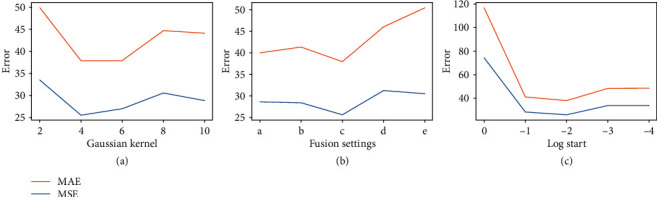
Sensitivity results of model parameters. (a) Adaptation of the Gaussian kernel. (b) Performance of different choices of multilayer fusion. Different structure settings are shown in Table 3. (c) Sensibility testing for the start in the log space.

*(1) Gaussian Kernels*. Here, we show the effect of different choices of Gaussian kernels. Five different Gaussian kernels *σ* = ({2, 4, 6, 8, 10}) are compared. The results shown in Figure 7 demonstrate that, unless the Gaussian kernel is set to be too small (*σ* = 2), the performance will not change dramatically. If the kernel size is too small, the generated Gaussian kernel covers only limited areas such that only a few pixels in the image have responses, which may exclude part of the plant root and confuse the network. Moreover, the error increases with increased kernel sizes. This is because the rice root only occupies few pixels. Large kernels lead to wrong labels of the background. Since *σ* = 4 obtains the best result compared with other choices, we fix *σ* = 4 in the following experiments.

*(2) Fusion Layers*. Here, we evaluate different choices of fusing layers. Since our multilayer fusion module can be applied to each layer, we report the performance of different fusion strategies. Following [26], the step of the sampling patch is set to 8 and the sampling size is set to 32. Note that different layers have different downsampling rates, and the steps and kernel sizes of average pooling are changed conditioned on the feature maps used. For the fusion choice that employs layer 5 as the output feature map directly, the redundant sampling step is set to 32. For fusing layers of 5 and 4, the step is 16. This is because their downsampling rates of feature maps are larger than 8 (32 and 16, respectively). The parameters of average pooling for different fusion choices are shown in Table 3, and their performances are shown in Figure 7. We can see that fusing 3 layers outperforms other choices. Compared with fusing 1 or 2 layers, fusing 3 layers receives more low-level details. However, fusing extra low-level features may weaken high-level semantic information; thus, fusing 4 and 5 layers increases errors. Since fusing 3 layers obtains the best result, we adopt this choice in the rest of the experiments.

*(3) Count Intervals*. Here, we show the effect of hyperparameters in classification. First, we evaluate five different choices of the starting point in the log space (*q* = {0, −1, −2, −3, −4}), and the results are shown in Figure 7.

The results demonstrate that our method is not sensitive to this parameter except when it is set to 0. This is because the patches with counts between 0 and 1 (*e*^0^) are significantly more than those in other counting intervals. If these patches are divided into one interval, the model will suffer from serious class imbalance. Since *q* = −2 reports the best results, we choose it as the default parameter.

We also verify the sensitivity of the quantization step *s* in the log space. The results shown in Table 4 demonstrate that our method is not sensitive to this parameter. We hence choose *s* = 0.1 as the default parameter.

#### 4.2.4. Effectiveness of Network Modules

Here, we verify the effectiveness of each module (MixNet-L backbone, MFM, and blockwise classification) in SFC^2^Net in Table 5. The baseline (without MixNet-L, MFM, and blockwise classification) shown in row 1 represents a model with the VGG16 backbone and local count regression. The results show that each module has a positive effect on performance improvement. Particularly, the network with only blockwise classification (row 4) achieves performance comparable to that of the full model, which demonstrates the surprising effectiveness of blockwise classification for rice plant counting.

#### 4.2.5. Inference Time

Here, we report the running time of SFC^2^Net on a platform with RTX 2060 6GB GPU and Intel i7-9750H CPU. The results of four random inputs of size 640 × 480, 1080 × 720, 1024 × 1024, and 1920 × 1080 are shown in Table 6. We also report the running time of CSRNet [22] and BCNet [24]. We observe that SFC^2^Net is fast when dealing with 1080 × 720, 1024 × 1024, and 1920 × 1080 images. However, it is slightly slower than BCNet [24] when dealing with a 640 × 480 input. We believe that the reason is that the advantage of depth-wise convolution is not fully exploited in low-resolution inputs. However, since images are usually of high resolution in agriculture, SFC^2^Net shows a clear advantage over its competitors in processing high-resolution images for high-throughput phenotyping.

### 4.3. Comparison with State-of-the-Art Methods

**Figure 8:**
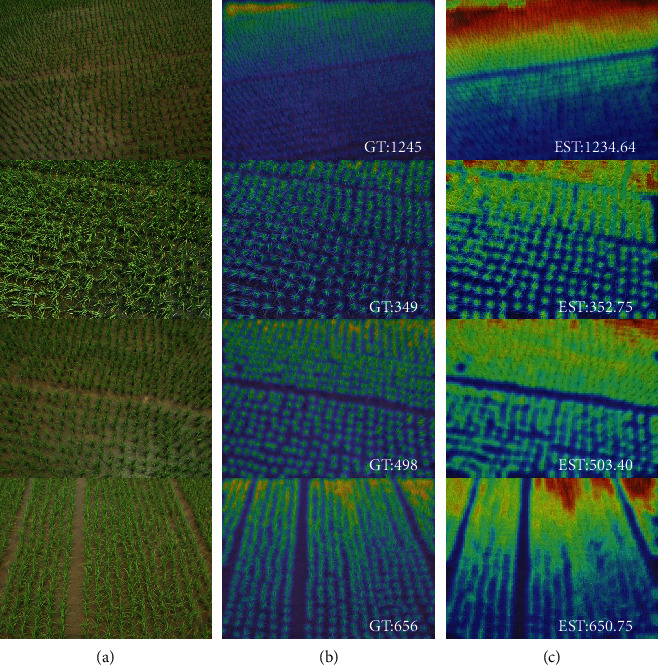
Qualitative results of our method. (a–c) The testing image, ground truth count map, and inferred count map. “GT” is the ground truth count and “EST” the estimation result.

**Figure 9:**
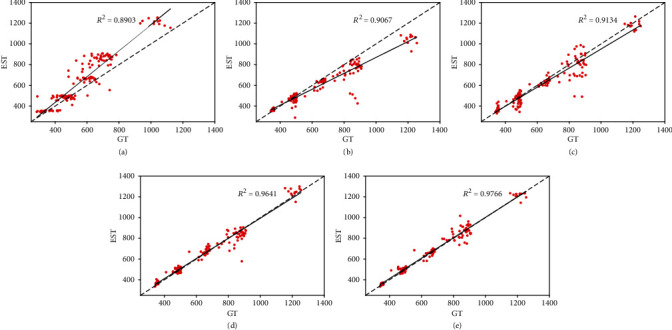
The coefficients of determination of five methods on the RPC dataset. “GT” denotes the ground truth results and “EST” the estimated results. (a–e) MCNN [21], TasselNetv2 [44], CSRNet [22], BCNet [24], and SFC^2^Net.

In this section, we compare our method with other state-of-the-art methods. MCNN [21] employs a multicolumn structure to extract multiscale feature maps to address scale variations. TasselNetv2 [44] proposes a fully convolutional structure to generate a redundant count map for wheat spike counting. CSRNet [22] utilizes VGG16 [33] as its backbone and replaces its final fully connected layers with dilated convolution to increase the receptive field. BCNet [24] transforms counting from a regression task into a multiclass classification problem to alleviate sample imbalance. The results shown in Table 7 demonstrate that our method outperforms these competitors and reduces the MAE and MSE by at least 18% and 23%, respectively. The qualitative results are shown in Figure 8, and the coefficients of determination of different methods are shown in Figure 9.

We also test our method on the MTC dataset [26]. The MTC dataset is created for maize tassel counting with 361 images collected from 16 imaging sequences. We compare SFC^2^Net with the existing methods that have reported their performance on this dataset. The results shown in Table 8 demonstrate that SFC^2^Net reports the new state-of-the-art performance. This experiment justifies the generality of our method for other plant species.

### 4.4. Failure Case Analyses

**Figure 10:**
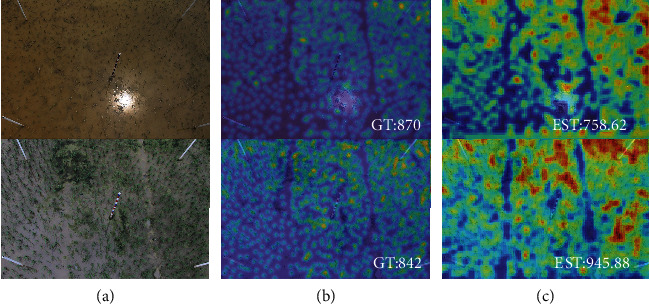
Failure cases: (a–c) the testing image, ground truth count map, and inferred count map. “GT” is the ground truth count and “EST” the estimation result.

In this section, we analyze some failure cases. Two examples are shown in Figure 10. Compared with other results, our model reports relatively large errors on these failure cases. We think the reason is that the rice plants in these cases have significantly different appearances with tiny leaves, and some of them look like a single point on the paddy field. On the contrary, the majority of plants in the dataset are with long leaves. It is worth noting that these failure cases all come from the images captured from Guangxi where images show obviously different appearances from the images captured in Jiangxi. The failure might be due to two reasons: either the diversity of the training dataset has to be improved or the method needs to be improved on the robustness to appearance.

## 5. Discussion and Conclusion

In this paper, we propose the deep learning-based network SFC^2^Net for rice density estimation. SFC^2^Net integrates the advantages of the mainstream object counting methods. With powerful feature representation and redundant blockwise classification, it improves the robustness to appearance variations and ameliorates sample imbalance. In addition, we collect a RPC dataset with 10 sequences between 2010 and 2013 in the rice field. A total of 211,971 dot annotations are manually labeled on rice plants.

In experiments, we empirically verify the influence of hyperparameters (Gaussian kernel and classification parameters), fusion decoder designs, and the counting by a classification paradigm with the MixNet-L backbone. The results show that (i) SFC^2^Net is not sensitive to the hyperparameters chosen, (ii) the multilayer fusion module can supplement details from low-level features and improve the performance, (iii) the introduction of the blockwise classification counting and MixNet-L backbone can significantly reduce the counting errors, (iv) SFC^2^Net is efficient, and (v) SFC^2^Net also outperforms state-of-the-art counting methods and reports highly accurate counting performance on the RPC dataset with a coefficient of determination of 0.98.

Although our method performs well on the test dataset, there still exists limitation waiting for further improvement. First, as per failure case analyses, our method still reports a relatively large error in some samples (about a 100 absolute error). This suggests that our method may have poor adaptation to other rice cultivars with significant differences in appearance.

Second, SFC^2^Net employs a blockwise classification counting method to ameliorate the sample imbalance during training. Indeed, it reports better results according to the ablation study. However, as discussed in [24], quantization errors exist in blockwise classification. When the accuracy of blockwise classification estimation surpasses a certain precision, the major error will lie in quantization errors.

Third, the RPC dataset certainly does not cover the whole data distribution of rice plants. In a real-world setting, the weather is susceptible to change. In the RPC dataset, the majority of samples were captured under nonrain conditions. Thus, the adaptation of the method to weather variations may be limited. Besides, the RPC dataset consists of the images from transplantation to tillering stages, which are only a part of rice growth. The CCD camera in the image-capturing device is also fixed with similar height and inclined angle, which implies that the model trained on the RPC dataset cannot deal with other application scenarios such as monitoring from hand-held smartphones or drones.

In future work, we will continue enriching the RPC dataset to adapt to different weathers, growth stages, perspectives, and rice varieties. Moreover, in this work, we only test our method on the precollected dataset. To deploy it in a real-world setting, we plan to test other flexible platforms.

## Figures and Tables

**Table 1:** Performance comparison between regression and classification.

Method	MAE	MSE	rMAE	*R* ^2^
Regression	34.58	55.61	5.27%	0.95
Classification	25.51	38.06	3.82%	0.98

**Table 2:** Backbone effectiveness verification.

Backbone	MAE	MSE	rMAE	*R* ^2^
VGG16	30.67	57.53	4.51%	0.95
MixNet-L	25.51	38.06	3.82%	0.98

**Table 3:** Parameters of average pooling for different fusion choices. “stride-a” and “size-a” are the stride and kernel size of average pooling, respectively, and “stride-s” and “size-s” are the stride and size of a sampling patch, respectively.

Setting	Fusion layer	Stride-a	Size-a	Stride-s	Size-s
a	5	1	1	32	32
b	5 + 4	2	1	32	16
c	5 + 4 + 3	4	1	32	8
d	5 + 4 + 3 + 2	8	2	32	8
e	5 + 4 + 3 + 2 + 1	16	4	32	8

**Table 4:** Sensibility testing for the step in the log space.

*s*	MAE	MSE	rMAE	*R* ^2^
05	30.06	54.81	4.38%	0.95
10	25.51	38.06	3.82%	0.98
15	28.98	41.02	4.87%	0.98
20	32.17	46.42	5.17%	0.97

**Table 5:** Effectiveness of the network module.

Modules			Metric			
MixNet-L	MFM	Classification	MAE	MSE	rMAE	*R* ^2^
×	×	×	51.41	85.40	7.45%	0.89
✓	×	×	46.88	68.44	6.96%	0.94
×	✓	×	47.61	85.94	6.75%	0.89
×	×	✓	31.28	49.82	4.76%	0.96
✓	✓	×	34.58	55.61	5.27%	0.95
✓	×	✓	28.62	39.98	4.74%	0.97
×	✓	✓	30.67	57.53	4.51%	0.95
✓	✓	✓	25.51	38.06	3.82%	0.98

**Table 6:** Inference time (frames per second) of different models.

Model	640 × 480	1080 × 720	1024 × 1024	1920 × 1080
CSRNet [22]	20.34	8.17	6.08	3.06
BCNet [24]	29.90	11.77	8.82	4.37
SFC^2^Net	22.70	19.40	16.70	8.68

**Table 7:** Comparison with state-of-the-art methods.

Method	MAE	MSE	rMAE	*R* ^2^
MCNN [21]	92.11	121.52	15.33%	0.89
TasselNetv2 [44]	59.39	95.80	7.86%	0.91
CSRNet [22]	49.22	74.58	7.47%	0.91
BCNet [24]	31.28	49.82	4.76%	0.96
SFC^2^Net	25.51	38.06	3.82%	0.98

**Table 8:** Performance comparison on the MTC dataset.

Method	MAE	MSE
JointSeg [47]	24.2	31.6
GlobalReg [48]	19.7	23.3
mTASSEL [49]	19.6	26.1
DensityReg [20]	11.9	14.8
CSRNet [22]	9.4	14.4
TasselNet [26]	6.6	9.6
SDCNet [35]	5.6	9.1
BCNet [24]	5.4	9.6
TasselNetv2 [44]	5.3	9.4
SFC^2^Net	5.0	9.4

## Data Availability

The RPC dataset has been made available at https://git.io/sfc2net.
